# P-141. Socio-Ecological Associations with Detectable Viremia among Pregnant Patients Living With HIV In South Brazil

**DOI:** 10.1093/ofid/ofae631.346

**Published:** 2025-01-29

**Authors:** Christopher J Hernandez, Fernando Echegaray, Mary C Cambou, Karin Nielsen-Saines

**Affiliations:** David Geffen School of Medicine, Los Angeles, California; David Geffen School of Medicine, Los Angeles, California; David Geffen School of Medicine University of California, Los Angeles, Los Angeles, California; David Geffen UCLA School of Medicine, Los Angeles, CA

## Abstract

**Background:**

Pregnant patients living with HIV are a priority group for the recruitment to the HIV healthcare cascade as uncontrolled HIV infection can confer an increased risk for adverse maternal and neonatal health outcomes and the risk for vertical transmission. Understanding the structural, interpersonal, and individual factors that are associated with detectable HIV viremia is of importance to guide outreach and intervention priorities.

**Figure 1.**
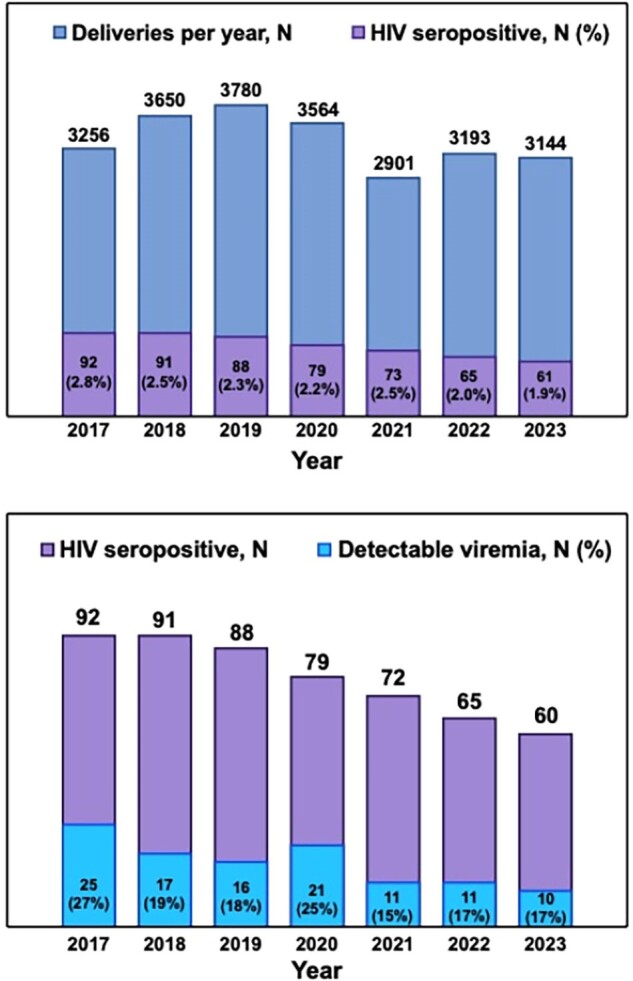
HIV prevalence and detectable viremia among pregnant patients delivering at a tertiary healthcare center in south Brazil.

Figure 1a. HIV seropositivity among pregnant patients hospitalized for delivery and figure 1b. Rate of detectable viremia by year between 2017 - 2023

**Methods:**

This was a retrospective cohort study of pregnant patients living with HIV who delivered from January 1, 2017, to December 31, 2023, at a tertiary-level hospital and referral institution for HIV care in Porto Alegre, Brazil. Detectable viremia was defined as 200 copies/ml at the time of delivery. The Socio-Ecological framework was used to guide hypothesis testing regarding associations with detectable viremia.

**Table 1.**
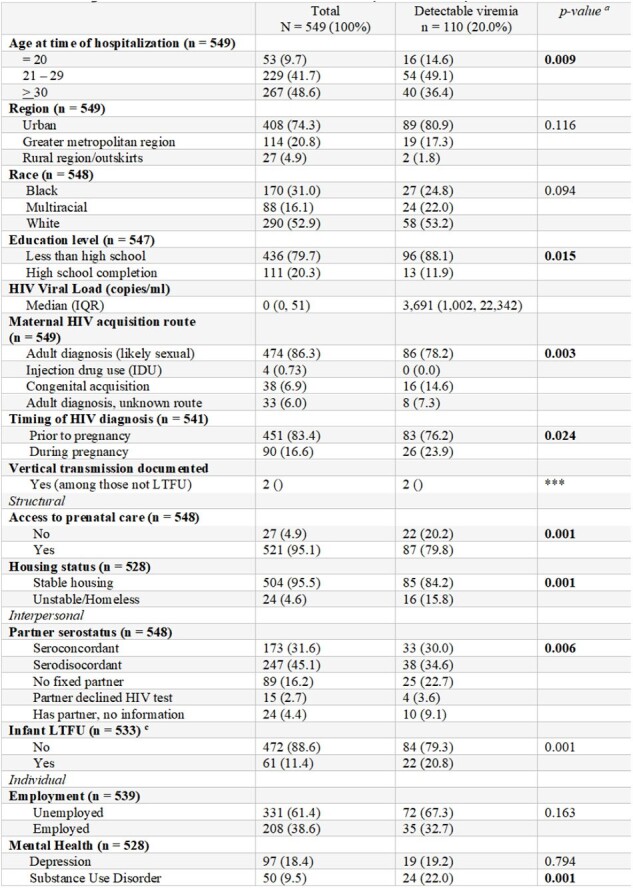
Demographics and clinical history of pregnant women living with HIV who delivered at Conceição from 2008 – 2018 (n= 549). a. Fisher’s exact tests were conducted if any one cell ≤ 5, otherwise Chi-square tests were used. b. Bipolar personality disorder, cognitive impairment, and other mental health conditions collapsed for analysis. c. The sixteen infant mortalities prior to follow-up completion period were dropped from analysis. *** N too small for statistical analysis.

**Results:**

In total, 549 patients were included, of whom 110 (20%) were found to have detectable viremia. Significant differences between detectable and undetectable viremia included ages 21 to 29 years (49.1% vs. 39.9%, 0.009), completing less than high school (88.1% vs. 77.6%, p = 0.015), congenital acquisition of HIV infection (14.6 vs. 5.0%, p = 0.003), seroconversion during pregnancy (23.9% vs 14.8%, p = 0.024), lack of prenatal care (20.2% vs. 1.1%, p = 0.001), homelessness (15.8% vs 1.9%, p = 0.001), serodiscordant partner (34.6% vs 47.7%, p= 0.006), infant loss to follow up (20.8% vs 9.1%, p = 0.001), substance use disorder (24.0% vs 6.1%, p = 0.001), crack use (19.5% vs. 7.1%, p = 0.001), and cocaine use (15.7% vs 7.1%, p= 0.001). Multivariable associations included prenatal care (adjusted Risk Ratio [aRR] = 0.20, 95% Confidence Interval [95% CI] = 0.15 – 0.26), homelessness (aRR = 4.02, 95% CI = 2.74 – 0.26), infant loss to follow up (aRR = 2.23, 95% CI = 1.51 – 3.29), substance use disorder (aRR =3.30, 95% CI = 2.23 – 4.87), lifetime use of crack (aRR = 2.82, 95% CI = 1.85 – 4.29), and cocaine (aRR = 1.89, 95% CI = 1.17 – 3.06).

**Table 2.**
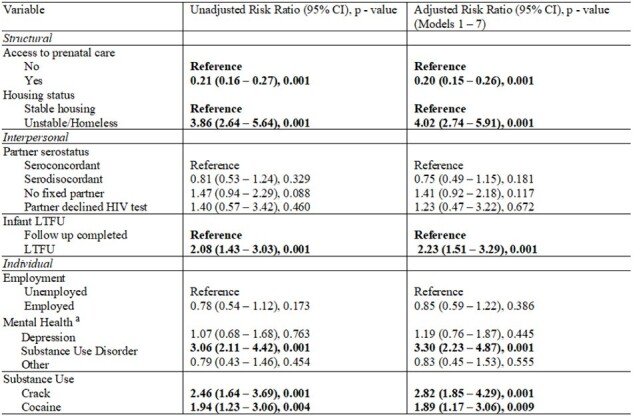
Left: Bivariate associations between socio-ecological factors and detectable HIV viremia. Right: Adjusted risk ratio associations between socio-ecological factors and detectable HIV viremia.

All models were adjusted by age, educational level, timing of HIV diagnosis, and maternal HIV infection acquisition route.

Model 1: Association of access to prenatal care on detectable viremia

Model 2: Association of employment on detectable viremia

Model 3: Association of housing status on detectable viremia

Model 4: Association of partner serostatus on detectable viremia

Model 5: Association of infant LTFU with detectable viremia.

Model 6: Association mental health on detectable viremia

Model 7: Association of crack on detectable viremia

Model 8: Association of cocaine on detectable viremia

**Conclusion:**

Intervention research should focus on housing and mental health services, with an emphasis on substance use treatment. Further, efforts to increase infant loss to follow-up should be integrated with maternal care as these are the patients with greatest risk.

**Disclosures:**

**All Authors**: No reported disclosures

